# Modelling *Toxoplasma gondii* infection in a 3D cell culture system *In Vitro*: Comparison with infection in 2D cell monolayers

**DOI:** 10.1371/journal.pone.0208558

**Published:** 2018-12-06

**Authors:** Jeffrey J. Danielson, Nicolas Perez, Julia D. Romano, Isabelle Coppens

**Affiliations:** 1 Department of Molecular Microbiology and Immunology, Johns Hopkins University Bloomberg School of Public Health, Baltimore, MD, United States of America; 2 Department of Chemical and Biomolecular Engineering, Johns Hopkins University School of Medicine, Baltimore, MD, United States of America; University at Buffalo, UNITED STATES

## Abstract

Three-dimensional (3D) cell culture models bridge the gap between two-dimensional (2D) monolayer cultures and animal models. Physiologically relevant, 3D culture models have significantly advanced basic cell science and provide unique insights into host-pathogen interactions intrinsically linked to cell morphology. *Toxoplasma gondii* is an obligate intravacuolar parasite that chronically infects a large portion of the global human population. Our current understanding of *Toxoplasma* infection is largely based on 2D cell cultures, in which mammalian cells are grown on flat surfaces. However, 2D cell cultures may not recapitulate key conditions of *in vivo* infections as they introduce artificial pressures and tensions, which may subsequently alter infectious processes that are dependent on spatiality, e.g., invasion, replication and egress. In this study, we adapted a collagen-based 3D cell culture system to reproduce the 3D environment of *T*. *gondii* natural infections for investigation of the replication and egress of the parasite from the parasitophorous vacuole. Suspended in the 3D matrix, *Toxoplasma*-infected VERO cells have round morphology, as opposed to infected VERO cells in 2D monolayers. The doubling time of *Toxoplasma* in VERO cells within the matrix is comparable to that of parasites cultivated in VERO cell monolayers. In the absence of the pressure of flattened host cells grown in 2D cultures, the parasitophorous vacuole of *T*. *gondii* has a globular shape, with intravacuolar parasites distributed radially, forming 3D spherical ‘rosette’ structures. Parasites egress radially away from the ruptured host cell in 3D matrices, in contrast to *Toxoplasma* cultivated in 2D monolayer cultures, where the parasites escape perpendicularly from the flat surface below the host cells. These observations demonstrate the utility of collagen matrices for studying parasite modes of infection as these 3D assays more closely mimic *in vivo* conditions.

## Introduction

*Toxoplasma gondii* is an apicomplexan parasite that causes life-long chronic disease in humans [1] and life-threatening symptomatic disease in immunocompromised individuals placed who are at risk of necrotizing encephalitis [2, 3]. As an obligate parasite, *Toxoplasma* completes its life cycle within a suitable host, constituting virtually all warm-blooded animals [4]. Upon invasion of a mammalian cell, the proliferative form of the parasite forms a parasitophorous vacuole (PV) wherein it replicates until it egresses from the host cell. A better understanding of the processes involved in the intracellular life cycle of *Toxoplasma*, such as invasion, replication and egress, will aid in the discovery of novel drug targets.

Historically, investigations on *T*. *gondii* parasitism have been divided between *in vitro*, such as 2D cell cultures, and animal infection models. Infection of monolayers (2D) of mammalian cells plated in culture dishes represent a cheap and convenient system for viewing infected cells and performing controlled studies with limited variables. However, the structural constraints of growing cells on flat surfaces in culture dishes yield cells with artificial shapes and different architecture than in animal tissues; thus it does not recapitulate natural infections. Animal models are exploited as tools to investigate cyst burden and distribution in tissues, immune responses, and pathogenicity of chronic infections. Commonly, rodents are used to holistically study infections as these animal models reproduce the neurological symptoms associated with toxoplasmosis (reviewed in [5]). Genetically-crossed mice with altered immune systems have been developed to parse out immune pathways and responses. The pioneering of a bioluminescence system in *T*. *gondii* (luciferin/luciferase), in addition to other genetic modifications of the parasite, has allowed the monitoring of the spread of an active infection in living animals [6]. However, animal models are time- and money-intensive, they give very little experimental precision on individual infections or do not permit the examination of parasite-host cell interactions at the subcellular level.

Recently, a third option has emerged to study *Toxoplasma*: a three-dimensional (3D) cell culture system *in vitro* that bridges the 2D monolayer and whole animal methods. Culturing broad range of cell types in a 3D matrix mimics the morphological and functional features of cells and tissues *in vivo* and provide a physiologically relevant model system to investigate host-parasite interactions. The altered morphology of cells grown in 2D cultures as flat monolayers may likely impact the parasite and PV morphology, as a result of the mechanical forces acting on the infected cell and the pressure of the culture medium. Consequently, the organization of parasites within the host cell as well as the dynamics of host-parasite interactions may differ in a complex 3D complex versus a confined 2D system. To this point, it has been established that monolayers of homogenous cells have different RNA profiles regarding migration, adhesion, signaling and morphology than their 3D counterparts [7–11], therefore likely providing a different cellular environment during infection. *Toxoplasma* is notorious to recruit mammalian organelles to its PV, usurping the host cytoskeleton and subverting many host cell pathways (reviewed in [12–14]). Culturing mammalian cells in 3D has revealed a different spatial organization of organelles and the geometry of the nucleus from 2D monolayers, which has yielded unanticipated features in organellar contact sites, nucleo-cytoskeletal connections, membrane protrusions and transcription-active subnucleolar compartments [15–18]. These differences between 3D and 2D systems emphasize how critical is to study the host cell manipulations by *Toxoplasma* in a physiological environment that more closely mimics *in vivo* conditions.

In toxicology, 3D culture systems have been intensively adopted in the search for potential cancer drugs [19–21]. Cancer cells cultured in 3D systems respond to drugs more similarly to *in vivo* counterparts, in terms of drug sensitivity and mechanisms of drug resistance than cancer cells cultivated in 2D systems. Importantly for drug screening applications, 3D *in vitro* matrices recapitulate more closely the *in vivo* conditions for solute diffusion, cell architecture and cell polarity [22]. Correspondingly, a 3D reconstitution system could be advantageous in screening for antitoxoplasma compounds as one major flaw in the drug pipeline is the inefficacy of compounds during *in vivo* trials despite having promising cidal activities in 2D settings.

There are three main methods for the *in vitro* culture of cells in 3D environments: the rotating wall vessel (RWV) bioreactor, collagen-based extracellular-like matrices (ECM) and organoids from pluripotent stem cells. In the RWV bioreactor, cells are cultured on spherical beads and are constantly rotated in a vessel filled with culture medium [23–25]. This method more accurately recapitulates fluid shear stress, cellular differentiation and host-pathogen interactions. A RWV system has been used to examine how *T*. *gondii* accesses the fetal compartment during infection *in vivo* [7]. In this system, a co-culture model has been developed to induce the fusion of trophoblasts to more precisely mimic the key features of the placental tissue *in vivo*. While cultured trophoblasts in 2D environments are susceptible to *Toxoplasma* infection, the placenta 3D model has revealed that these cells, in fact, form an effective barrier to parasite invasion and are refractory to infection, even at high multiplicity-of-infections.

In 3D extracellular matrices, cells are suspended in collagen gels, fibrin gels, cell-derived matrix (CDM) composed of proteins, e.g., fibronectin produced naturally by fibroblasts, or basement membrane extract (BME or Matrigel) (summarized in [9]) to reconstitute the physical properties of an ECM. 3D matrices do not have fluid movements like the RWV bioreactor or *in vivo* environments, but are used to study cell attachment on all three axes and cell motility through the matrix [26]. A 3D Matrigel-based environment has been exploited to examine the properties of gliding motility of extracellular *Toxoplasma* [27]. On protein-coated glass coverslips, *T*. *gondii* engages a twirling, circular gliding and helical gliding, however, the parasite’s motility in a 3D Matrigel is strikingly different as *Toxoxplasma* moves in irregular corkscrew-like trajectories. Another study has used a collagen gel-based matrix to analyze the locomotion of *Toxoplasma*-infected dendritic cells (DC) [28]. On 2D surfaces, infected DC, as opposed to uninfected DC, exhibit a hypermotility phenotype that is based on integrin-dependent adhesion [29]. In 3D collagen gels, infected DC show significantly enhanced penetration of the matrix compared to bystander uninfected DC, but they exhibit an integrin-independent, amoeboid-like hypermigratory phenotype, thus demonstrating the utility of 3D models for studying parasite modes of infection.

In organoids, stem cells are differentiated to recreate crucial aspects of the architecture, cellular composition and function of *in vivo* tissues [30]. Intestinal and lung organoids have been successfully generated to study the apicomplexan parasite *Cryptosporidium parvum*, a causative agent of diarrhoeal and respiratory diseases in humans, for which no optimal *in vitro* culture system exists. Monolayers of primary epithelial cells only support short-term infection of *C*. *parvum*, with incomplete propagation and cell cycle progression [31]. In organoids, however, the entire life cycle of *C*. *parvum* can be completed. Intestinal organoids have been also successfully tested for *Toxoplasma* infection [32]. As the usual port of entry of *Toxoplasma* into its hosts is through the intestinal route via ingestion of cysts, enteroids offer a promising experimental system to recapitulate host-parasite interactions at the primary infection site.

In this study, we established a 3D microenvironment based a collagen matrix to examine the intracellular development of *Toxoplasma* in individual mammalian cells by microscopy, focusing on parasite replication, egress and intravacuolar organization.

## Materials and methods

### Reagents and antibodies

All chemicals were obtained from Sigma (St Louis, MO) or Thermo Fisher Scientific (Waltham, MA) unless otherwise stated.

### Culture conditions of mammalian cell lines and parasites

Monkey VERO cells and human foreskin fibroblasts (HFF) obtained from the American Type Culture Collection (Manassas, VA) were grown as monolayers and cultivated in α-minimum essential medium (MEM) supplemented with 10% fetal bovine serum (FBS), 2 mM glutamine and penicillin/streptomycin (100 units/ml per 100 μg/ml), and maintained at 37°C in 5% CO_2_. The tachyzoites from the RH strain (Type I lineage) were used throughout this study and propagated *in vitro* by serial passage in monolayers of HFF [33]. *Toxoplasma* stably expressing RFP (from RH strain) used in our fluorescence microscopy assays were generously provided by F. Dzierszinski (McGill University, Montreal).

### 3D matrix collagen protocol

HFF or VERO cell monolayers were washed with Hanks’ Balanced Salt solution (HBSS), trypsinized, counted and resuspended in 50% α-MEM and 50% reconstitution buffer (200 mM NaHCO_3_, 250 mM HEPES; pH 7.2). Cells in suspension were then mixed with a solution of Type I collagen from rat tail (Corning High Concentration Collagen) at a final concentration of collagen of 2 mg/ml. To neutralize the acidic properties of collagen, 1 M NaOH was added to restore the pH to 7.2. The mixture was gently pipetted up and down several times to homogenize the solution, and a volume of 500 μl of cells in non-polymerized collagen was placed into 8-well chambered slides. The plate was set in a CO_2_ incubator at 37°C to induce collagen polymerization for at least 1 h and could be kept in the incubator for up to 3 days.

### 3D matrix mammalian cell viability assays

A Trypan blue exclusion test was used to assess VERO cell viability during 3D cultivation. The collagen matrices with VERO cells in suspension were fixed for 10 min with 4% paraformaldehyde (PFA) and stained for 10 min with 0.1% Trypan Blue before washing with PBS and observation by microscopy. As a control, 2D VERO monolayers were PFA-fixed and stained for 1 min with 0.1% Trypan Blue. At least 1,000 cells were counted for each condition.

### 3D matrix parasite replication assays

VERO cells in 2D monolayers were infected with RFP-expressing *T*. *gondii* for 20 min, trypsinized and resuspended in a 3D collagen matrix. After 24 h, the matrices were fixed with 4% PFA and visualized under the microscope to enumerate the parasites per vacuole at 24 h post-infection (p.i.). As controls, infected trypsinized VERO cells were seeded in a 24-well plate or were set on top of a thin layer of polymerized collagen as a substrate placed in the bottom of a 24-well plate. At least 800 and 1,200 vacuoles were counted per 3D and 2D conditions, respectively.

### 3D matrix parasite egress assays

VERO cells in 2D monolayers were infected with RFP-expressing *T*. *gondii* for 10 min, trypsinized and resuspended in a 3D collagen matrix for 48 h to 60 h. To capture egress, infected cells were individually found and centered in the field of view of a 20x objective. The camera was set to take phase-contrast image of the cells and RFP-channel images every 1 min for 12–15 h with *z*-slices ~20 μm apart to cover the cell from the bottom to top.

### Fluorescence microscopy

3D images of live or PFA-fixed cells were acquired at 20x with a Nikon TE2000 microscope with a Luca-R EMCCD camera or at 40x with a Nikon A1 swept-field confocal microscope equipped with a DS-QiMc camera and motorized stage (Prior Scientific), both controlled by Nikon NIS Elements software. 2D images of live cells were captured at 40x with a Zeiss AxioImager M2 equipped with a Hamamatsu Orca-R2 digital camera and Volocity software. Images from cell monolayers were deconvolved using an iterative restoration algorithm (Volocity).

## Results and Discussion

### Optimization of a 3D collagen matrix system for intracellular *Toxoplasma*

Cell behavior in 2D monolayers and 3D matrices differ in cell signaling and biochemical properties, demonstrating the importance of 3D cellular dimensionality in mimicking physiological conditions [8–11]. As a major component of connective tissues *in vivo*, collagen I exists as fibers, and is the most widely used protein for *in vitro* 3D studies based on ECM [34]. We used collagen fibers to reproduce an ECM-like environment for the 3D cultivation system of cells infected with *T*. *gondii* to compare parasite processes in 3D and 2D cell culture models. First, we selected human foreskin fibroblasts (HFF) as host cells for *Toxoplasma* in our 3D cultivation system since HFF are routinely used in many laboratories for 2D culture as they grow as large and flat cells that are contact-inhibited. To create a 3D system, we disrupted HFF monolayers with trypsin treatment and suspended the HFF in a collagen solution at the final concentration of 2 mg/ml, which mimics the natural density of collagen in organs and tissues within animals. However, under these conditions (2 mg/ml of collagen), the vast majority of HFF sunk to the bottom of the well of a 24-well plate instead of being suspended in the matrix. We tested higher concentrations of collagen, in 0.5 mg/ml increments, and observed that HFF mixed with 4 mg/ml of collagen remained in suspension in the matrix (not shown). However, such a high collagen concentration was suboptimal to monitor dynamic interactions between the parasite and its host cell because HFF lost their motility and growth capacity.

**Fig 1:**
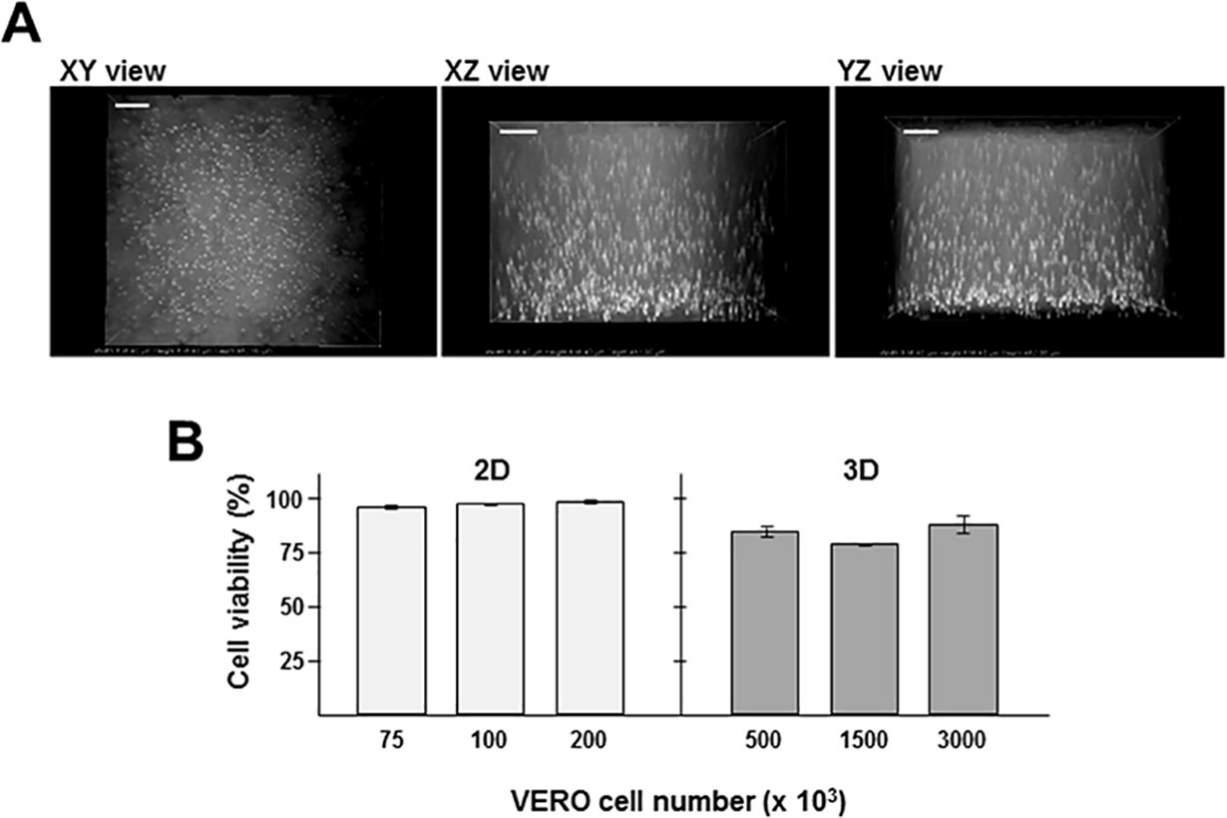
Representative image of VERO cells suspended in a 3D collagen matrix and cell viability. A. Microscopy observation of live cells at 20x showing dark field images of 3 views of a single 3D collagen matrix seeded with 500,000 VERO cells in suspension. The 3D image was reconstructed from images taken every 5 μm. A top-down (XY) view is shown as well as both side (XZ) and (YZ) views. Width and height of the model was 636.4 μm and the depth was 462.0 μm (scale bars, 80 μm). VERO cells were suspended at all levels within the matrix with no clumps of cells, with the exception of a few cells that sunk to the bottom of the well and attached to the plastic, but those cells were ignored for the processes of our experimentation. B. Comparison of VERO cell viability in 3D collagen matrix and 2D monolayer cultures. After 2D cultivation, trypsinized VERO cells were suspended in 3D matrices or placed in the bottom of a 4-well plate for 24 h prior to fixation and staining with Trypan Blue. Date are means ± SEM, from 3 independent biological samples made in triplicate. Cell viability was similar at comparable cell density conditions (NS; p ≤ 0.1434 with two-tailed t-test).

To overcome the limitation of collagen concentration for HFF used in our 3D system, we tested epithelial VERO cells, which are larger in size and volume than HFF. VERO cells have also been used for *T*. *gondii* experimentation in 2D monolayers, due to their resistance to senescence and amenability for genetic manipulations, allowing the generation of mutant host cells to study *Toxoplasma* infectivity. Unlike HFF, most VERO cells remained in suspension throughout a matrix composed of 2 mg/ml of collagen, as visualized by microscopy in XY, XZ and YZ axes (Fig 1A). Cellular morphology differs between cells grown in 3D matrices versus on a 2D conditions (plastic or glass support) as stiff substrates induce the formation of focal adhesions and stress fibers [9]. In accordance with the literature, we observed that VERO cells cultured in 3D were rounder, with fewer lateral protrusions than VERO cells grown as 2D monolayers (not shown).

Mammalian cell density affects *Toxoplasma* infection rates, with VERO cells confluency of 85% being optimal [35]. To directly compare *Toxoplasma* infections in 3D and 2D systems, we first determined the equivalency of VERO cell densities between in 3D and 2D cultures. To compare cell densities in 3D and 2D cultures, we titrated cells by serial dilution, assessed the number of cells per field of views and calculated the number of cells per volume. VERO cells, at various concentrations, were mixed with collagen or medium alone, and plated into the wells of a 24-well plate. After 24 h, the number of cells was assessed by microscopy. We calculated that 500,000 VERO cells in our 3D culture had a comparable density to 75,000 VERO cells in a 2D monolayer as the volume of the 3D matrix was greater than the surface area of the 2D culture.

We compared the viability of VERO cells grown in 3D collagen matrices and 2D monolayers by using a Trypan Blue exclusion assay. VERO cells from monolayers were dissociated with trypsin treatment and cultured at various cell concentrations, either as flat monolayers in plastic dishes or in suspension in collagen matrices for 24 h (Fig 1B). Regardless of initial plating densities, the viability of cells in 2D culture remained > 96% while in 3D culture they ranged from ~80 to 90%. For example, a viability of 96.6 ± 1% was measured for cells plated as the initial concentration of 75,000 number in 2D while a viability corresponding to 86.1 ± 4.4% was calculated for cells in the collagen matrix at the comparable density of 500,000 cells in 3D. The differences in cell viability between the 2D and 3D culture conditions, however, were not statistically significant.

### *Toxoplasma* cultivated in 3D collagen matrices and 2D cell monolayers have the same replication rate

Having optimized our experimental conditions for VERO cell cultivation in 3D collagen matrices (see technical details in Materials and Methods section), we sought to assess *Toxoplasma* infectivity from invasion through replication to egress in our new 3D system. In an initial scheme, we examined the ability of *T*. *gondii* to invade collagen-encapsulated VERO cells by introducing a suspension of parasites at a multiplicity-of-infection (MOI) of 3 to the top of the gel matrix. The collagen matrix may represent a physical hindrance for parasite motility as the pores of the matrix with a collagen concentration of 2 mg/ml have a mean diameter of 2 μm [36] and *Toxoplasma* measures is ~6 μm (long) by ~2 μm (wide). After three attempts, we did not detect parasites traversing the matrix to any discernable depth, and thus the VERO cells were never infected. To avoid these constraints, we mixed freshly egressed *Toxoplasma* with VERO cells (MOI of 3) in the presence of non-polymerized collagen for 10 min at room temperature before adding NaOH and placing cells in a CO_2_ incubator to induce collagen polymerization. However, this strategy resulted in very few infected cells after 24 h. Possibly, the cells and parasites were not in close enough proximity in the collagen matrix for efficient invasion and the matrix pores, with a mean pore size of 2 μm [36], continued to present an obstacle for parasite motility towards a host cell. As our attempts to infect VERO cells within the collagen matrix resulted in null to menial infection rates, we instead infected cells in a 2D monolayer prior to suspension in a collagen matrix. Monolayers of VERO cells were infected with added freshly egressed *T*. *gondii* (MOI of 3) at 37°C for 10 min., then the cells were dissociated by trypsin treatment and resuspended in a non-polymerized collagen solution. Then, the collagen containing the infected cells was allowed to polymerize in a CO_2_ incubator.

**Fig 2:**
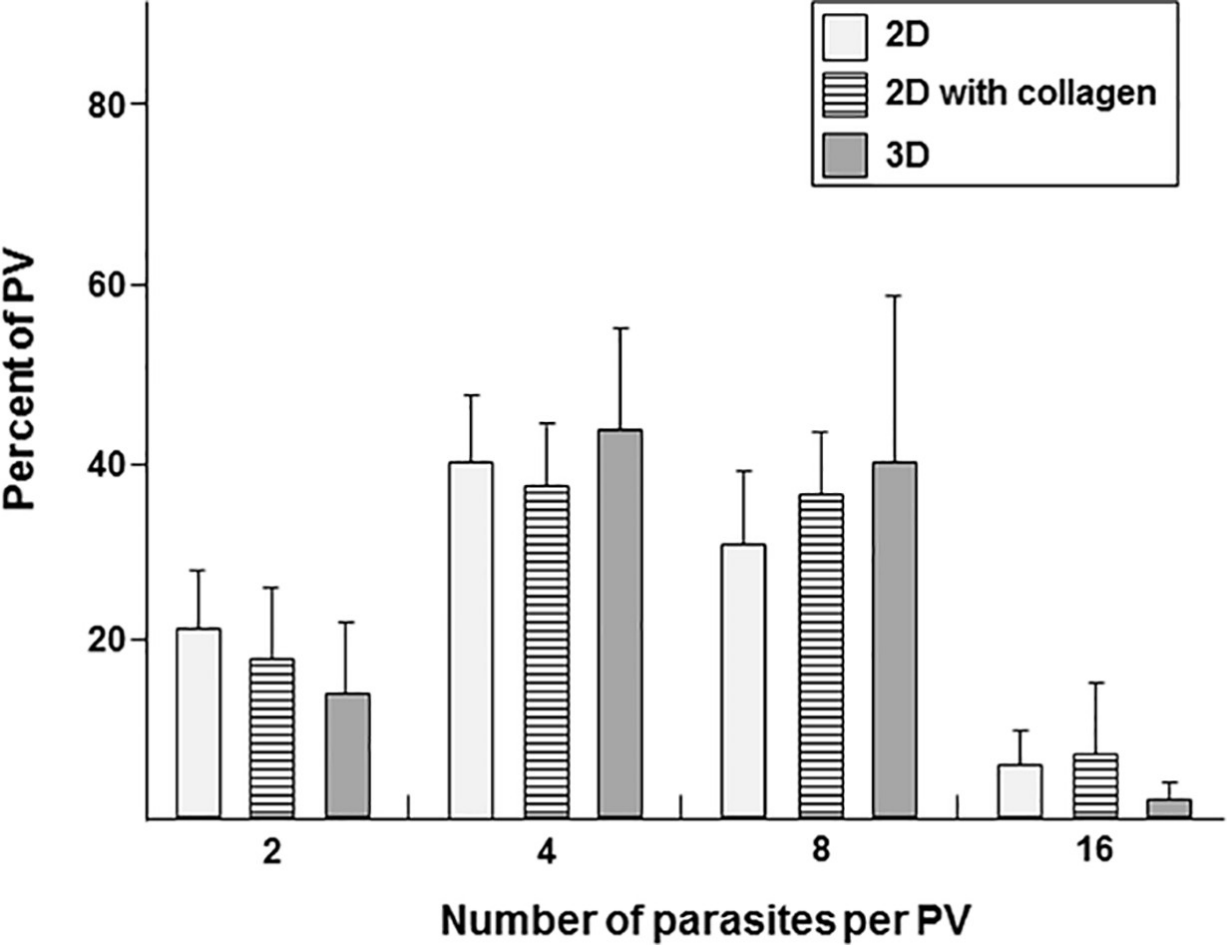
Comparison of parasite replication in 3D collagen matrix with 2D cell monolayers. After 2D cultivation, trypsinized VERO cells were suspended in 3D matrices or placed in the bottom of a 24-well plate either with or without collagen coating for 24 h prior to fixation and parasite counting. Date are means ± SEM, from 4 independent biological samples made in triplicate. No statistical differences between the 2D and 3D conditions were observed (NS; *p* > 0.05 with χ^2^ test).

We then examined the ability of *T*. *gondii* to multiply in VERO cells suspended in a 3D collagen matrix. During asexual reproduction, *T*. *gondii* replicates by endodyogeny, a process in which two daughter cells are simultaneously produced inside a mother cell, which will be consumed by the daughter cells before their escape from the ruptured mother cell [37]. VERO cells infected with RFP-expressing *T*. *gondii* were added to a 3D collagen matrix as described above, seeded to a 24-well plate (plastic support; 2D) or placed on top of a thin layer of polymerized collagen (collagen support; 2D). After 24 h, parasite replication was quantified by enumerating parasites per PV. No difference in replication rate was observed for parasites developing in cells in the 3D matrix versus 2D monolayers, either without or with a thin collagen layer (Fig 2). The replication rate of parasites in each condition was extrapolated using the formula {*Toxoplasma*_initial_} _*_ 2^x^ = {*Toxoplasma*_final_} based on the number of parasites per PV, with the variable ‘x’ representing the parasite doubling time. The averaged time of *Toxoplasma* replication was 9.6 h in 3D matrices, as compared to 9.3 h and 9.8 h in 2D monolayers with and without collagen, respectively.

### *Toxoplasma* forms a spherical PV with parasites organized in a tridimensional ‘rosette’ within cells in a 3D collagen matrix

**Fig 3:**
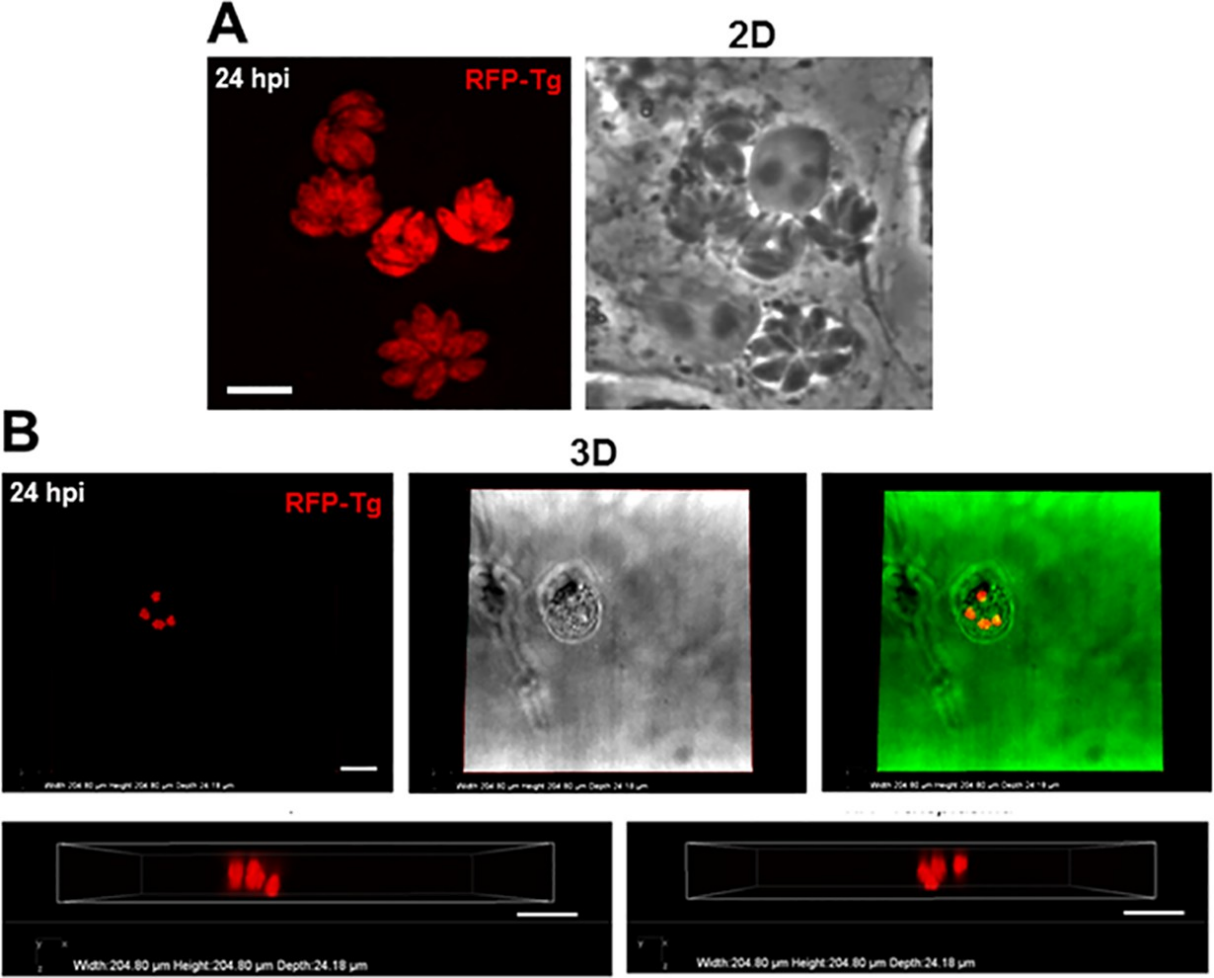
Morphology of infected cells and PV within VERO cells in 3D collagen matrix. A. Fluorescence microscopy at 40x of live multi-infected VERO cells with RFP-expressing *Toxoplasma* grown in 2D culture for 24 h showing flat rosette formation. Scale bar, 10 μm. B. Fluorescence microscopy at 40x of live multi-infected VERO cell with RFP-expressing *Toxoplasma* grown in 3D culture for 24 h (left), bright field image (middle) and overlay image that have been pseudo-colorized in green to increase contrast. The top row shows a top-down view and the row below two side-on views. Images reveal 4 globular PV at different depths within a single cell. Scale bar, 25 μm.

In 2D cell monolayers, the parasites are arranged within the PV in a radial structure or ‘rosette’, with their apical end facing the PV membrane and their basal ends attached to the residual body, a structure containing remnants of the mother cell, at the center of the PV [38]. This polarized arrangement of *Toxoplasma* is hypothesized to influence movement of nutrients from the host cell and export of parasite antigens into the host cell. The ‘rosette’ intravacuolar arrangement of *Toxoplasma*, as illustrated in Fig 3A is clearly visible in 2D culture conditions, as the PV forms a ‘dome-like structure’, with the flat side facing the bottom of the culture dish. The ‘rosette’ spatial organization in a semi-flat PV, however, may be an artifact of the stress forces imposed on 2D cell cultures. In a 3D matrix in which the pressure on cells and stress forces would be lessened, we observed that the shape of the PV was globular, as illustrated in the infected VERO cell in panel be of Fig 3B showing top and lateral views of 4 PV.

**Fig 4:**
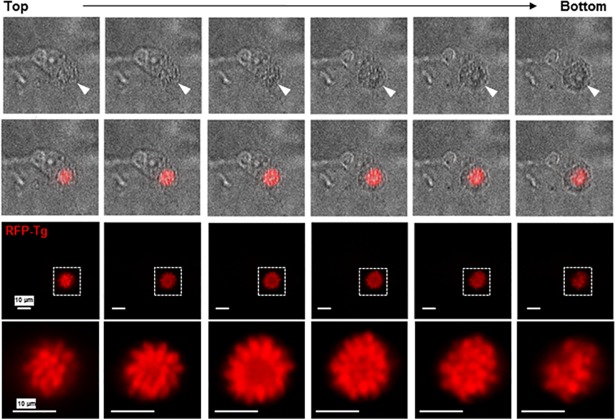
Organization of the parasites in PV within VERO cells in 3D collagen matrix. Microscopy observation at 40x of a live VERO cell infected with RFP-expressing *Toxoplasma* and cultivated in a 3D collagen matrix for 24 h (XY views). Upper panels show bright field images, with arrowheads pinpointing the PV, lower panels illustrate RFP-parasites by fluorescence, and middle panels are overlay images. Insets show the PV at 3.5x magnification. Images were taken every 1 μm in depth and reveal symmetrical organization of intravacuolar *Toxoplasma*, comparable to rosette-like structures formed by parasites within a PV.

Within these globular PV, the parasites were radially distributed as in 2D culture, but formed a spherical ‘rosette’ structure, with one end of each parasite pointing toward the PV membrane, as shown in a *z*-series of a single rosette within an infected VERO cells suspended in a collagen matrix (Fig 4). The averaged diameter of the PV was 18 ± 3 μm^2^ at 24 h (n = 13 PV). Jointly, these observations reveal a sphere-shaped PV and ‘rosette’ structures of parasites in infected cells cultivated in 3D collagen matrices, as opposed to 2D monolayers. These more-rounded morphological features are presumably closer to *in vivo* PV phenotypes as there would be fewer stresses than in a 2D system. The spherical nature of the PV and ‘rosettes’ may favor the intracellular development of *Toxoplasma* by maximizing the parasite’s interactions with the host cell due to increased PV surface area.

### *Toxoplasma* egress from cells in the 3D collagen matrix radially in all directions

**Fig 5:**
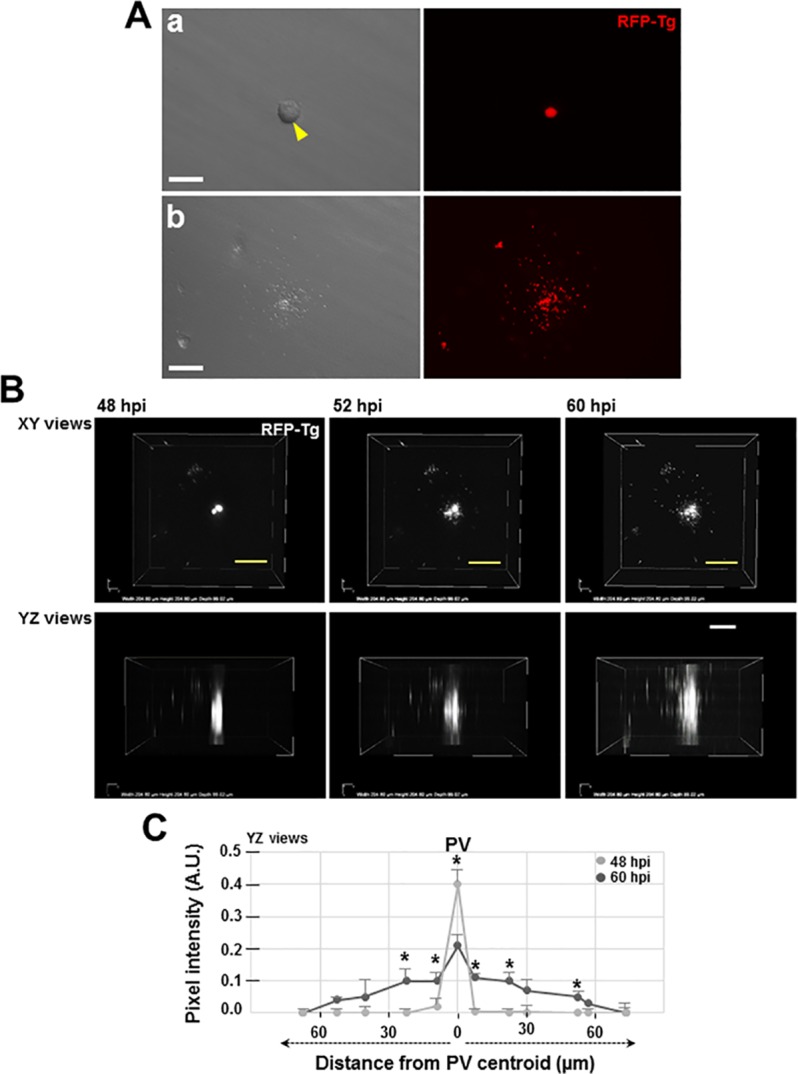
*Toxoplasma* egress from VERO cells in 3D collagen matrix. A. Microscopy images at 20x of a VERO cell infected with RFP-expressing *Toxoplasma* (RFP-Tg) for 60 h in a 3D collagen matrix (XY view), showing a representative image of the same PV just before (panel a) or after rupture with parasites escaping in various directions (panel b). Left: phase-contrast images; right: fluorescence microcopy. Arrowhead shows the infected cell from where the parasites escaped. Bar, 40 μm. B. Live fluorescence microscopy of a VERO cell multi-infected with RFP-expressing *Toxoplasma*, in a 3D collagen matrix, at the indicated times p.i. Images show top-down (XY) and side-on (YZ) views of the infected VERO cells over the course of parasite egress. After 52 h of infection, individual parasites escape the ruptured cell in all directions and at 60 h p.i. a more diffuse fluorescence signal appears concomitant with parasite dispersion in the matrix as they move away from the former host cell. Scale bars, 50 μm. C. Quantification of fluorescence intensity of RFP-expressing *Toxoplasma* within the PV (48 h p.i.) or during egress (60 h p.i.). Regions-of-Interest corresponding to the fluorescence signal of parasites were drawn at the indicated distances from the ruptured cell to collect numbers that are integrated pixel intensity of entire area minus background using the ImageJ analysis program. Data are means of three independent biological experiments (n = 4 to 9 PV) showing statistical differences between distances from the PV centroid at 48 and 60 hpi (*, p < 0.05 with two-tailed t-test).

After several cycles of replication, the PV occupies a large territory of the host cytoplasm, and *Toxoplasma* need to leave the host cell to invade nearby mammalian cells to start the cycle anew [37]. Egress of *Toxoplasma* is characterized by the disassembly of the ‘rosette’, active disruption of the PV membrane, breakdown of the host cell plasma membrane, and finally release of free parasites in the medium. Egress is triggered by parasite secretions and vacuole acidification (reviewed in [39]). In movies of egressing parasites from 2D cell monolayers, the parasites escape from cells perpendicularly from the hard dish surface below the cells. To gain insight on the egress events of parasites from cells in 3D matrix, we imaged live cells infected with RFP-expressing *Toxoplasma* until egress events occur (Fig 5A). We showed that egressed parasites 6o h p.i. were distributed all around the ruptured host cells, suggesting egress events in multiple directions. To verify this possibility, we performed live imaging on infected cells by focusing on a cell containing two large PV and setting the camera to take pictures at several *z*-depths every 5 minutes from 48 h to 60 h p.i. (Fig 5B). The parasites rapidly leaved the cell and, then spread out radially away from the ruptured cell. The fluorescent signal became then more diffuse in the matrix, concomitant with the dispersion of the parasites. Quantification of the fluorescence intensity in the 3D matrix at 48 h and 60 h show an almost symmetric signal, centered on the ruptured cell, confirming that the parasites were dispersing in all directions. Thus, the pressure and tension imposed on a PV in 2D cell monolayers probably do not reciprocate the situation *in vivo*, as the paths of egress differs between 2D and 3D environments.

## Conclusions

To investigate the program of infectivity of intracellular *T*. *gondii* in a more physiological *in vitro* environment than flat monolayers cultured on plastic dishes, we established a 3D cell culture system based on collagen I polymers. In our 3D culture system, infected cells had a rounder morphology, PV were more globular in shape and the parasites organized themselves into spherical ‘rosettes’ within the PV. In the 3D system, the replication rate of *Toxoplasma* was similar to that of parasites in 2D cultures, with an averaged doubling time of 9 h. The rounder morphology of the PV in 3D systems would likely result in a greater surface area for contacts between the PV membrane and host organelles than for the ‘dome’-shaped PV typical observed in 2D monolayers. Both in 2D and 3D cell culture, parasites egressed from host cells around 50 h p.i. However, in 3D culture systems, parasites escaped and moved radially away from the lysed cell in three-dimensions while in 2D systems the bottom of the plate presented a barrier, which may allow the parasite to explore vaster surroundings and find new host cells. Thus, 2D cell cultivation introduces non-physiological pressures and tensions that may alter some processes of *Toxoplasma* infection.

While culturing infected cells in 3D collagen systems may better mimic physiological conditions than in 2D monolayers, the exploitation of the 3D system to monitor *Toxoplasma* infection can be limiting. One major caveat is that imaging intracellular *Toxoplasma* in cells buried within a matrix at different depths is more challenging than in 2D monolayers and is limited to 40x magnification as the working distance of higher magnification objectives does not allow penetration into the matrix depth. Obviously, the change in morphology of infected cells in 3D culture to a more round and shrunken shape renders microscopic observations more complex than on flat, spread-out monolayers. Also, we were unable to establish a successful method for modeling invasion in our 3D environment likely due to the steric (i.e., porosity and/or fiber size) and mechanical (i.e., stiffness or compliance) properties of the collagen gel, which impedes parasite penetration into the matrix and, thus, invasion of mammalian cells.

Despite the shortcomings, valuable insights into single cell infection and host cell-parasite dynamics may be obtained using a 3D *in vitro* culture system. In fact, RNAseq studies have revealed differential gene expression in cells cultivated in 2D versus 3D systems [7]. It is therefore likely that parasite gene expression profiles may differ in a 3D environment and may account for differences in infectivity processes between cultivation in monolayers and *in vivo*. For instance, 3D environments may simulate nutrient gradients, which may induce gene expression changes for resource acquisition and response to differing external stimuli. Future work in this system should explore parasite infectivity at the subcellular level, such as examining whether *Toxoplasma* reorganizes the host cell interior and recruits host organelles in 3D cultured mammalian cells, as it has been established in infected cells cultured as 2D flat monolayers [14, 40]. Finally, a 3D collagen system may be useful for drug discovery and trials against *Toxoplasma*, as the system has been successfully implemented in cancer toxicology studies [19–21]. As 3D cell culture systems have shown better recapitulation of *in vivo* trials, they may streamline the process and save time and money. While 3D collagen matrices mimic more closely *in vivo* cellular morphology allowing for investigations of host-parasite interactions on individual cells, organoids better represent the architecture, cellular composition and function of *in vivo* tissues. In the future, combining studies using 3D extracellular matrices with those of organoids will provide insights into *in vivo Toxoplasma* infection modalities at the cellular and tissue levels.
